# Eco-friendly design inspired by nature: examining adolescents' perceptions of biomimicry

**DOI:** 10.3389/fpsyg.2026.1833615

**Published:** 2026-06-30

**Authors:** Sena Öz Tatacak, Hatice Zelal Bingöl, Figen Gürsoy

**Affiliations:** 1 Department of Child Development, Faculty of Health Sciences, Ankara University, Ankara, Türkiye; 2 Department of Child Development, Faculty of Health Sciences, Istanbul Gelisim University, Istanbul, Türkiye; 3 Department of Child Development, Institute of Health Sciences, Ankara University, Ankara, Türkiye

**Keywords:** biomimicry, connectedness to nature, nature-inspired innovation, science high school students, sustainable design

## Abstract

**Introduction:**

Interest in biomimicry education has grown recently, yet little is known about how high-achieving science high school students conceptualize biomimicry and translate biological knowledge into sustainable design ideas across disciplines.

**Methods:**

Using a qualitative case study design, data were collected from 29 ninth-grade students via a written semi-structured form and analyzed through content analysis using the Biology-to-Design Spiral as the analytical framework.

**Results:**

Students were familiar with biomimicry and successfully transformed biological knowledge into design logic, but their conception of “living things” remained predominantly animal-centric and the range of inspiration organisms was narrow despite daily contact with the school grove. Designs centered on structural and functional traits and were linked to technology-based, sustainability-oriented solutions.

**Discussion:**

Everyday contact with nature alone does not appear sufficient to broaden biomimicry thinking; structured learning environments connecting ecological experience with scientific and design reasoning are needed. Biomimicry education shows strong interdisciplinary potential to integrate biology, engineering, and sustainability and to foster ecological thinking among gifted adolescents.

## Introduction

The notion that “what works in nature can also work for humanity” introduces the concept of biomimicry (Karabetça, 2018). Rather than consuming nature as a mere resource, this approach centers on learning from it, aiming to adapt effective and enduring strategies discovered by organisms through millions of years of evolutionary processes into technology and design (Rao, 2014). Etymologically derived from the Greek words bios (life) and mimesis (imitation), biomimicry represents the conscious emulation of life's genius (Benyus, 1997).

For a design to be categorized within the scope of biomimicry, three fundamental elements must coexist: ethos (the intent and responsibility toward nature), emulate (the translation of biological principles and strategies into design), and re-connect (strengthening the relationship between humans and nature). The holistic integration of these elements distinguishes biomimicry from other bio-inspired approaches; if any element is absent, the design falls outside the strict definition of biomimicry (Biomimicry 3.8., 2015). Furthermore, the field operates through two primary methodological pathways: the “top-down approach (problem-driven),” where a designer seeks solutions in nature for a specific human problem, and the “bottom-up approach (solution-driven),” which involves developing new design solutions through the detailed analysis of biological structures and processes (Zari, 2007). This latter approach necessitates a deep investigation of biological mechanisms before they are funneled into a specific problem-solving context, embodying the “biology-to-design” philosophy. This cognitive framework is operationalized through the “Biomimicry Design Spiral,” (also referred to as the Biology-to-Design Spiral) a flexible process encompassing stages of discovering, abstracting, brainstorming, emulating, and evaluating (Macnab, 2012). This spiral allows researchers to initiate the design process at various stages, offering a versatile tool for creating sustainable and nature-friendly innovations (Purwaningsih et al., 2024). In this context, biomimicry is conceptualized not merely as a formal imitation of nature but as a strategic ‘thinking path' that transitions from nature-inspiration to emulation, ultimately serving as a vital methodology for achieving long-term sustainability goals (Oguntona and Aigbavboa, 2023).

As biomimicry positions nature as a “mentor” and reminds humanity of its place within the ecosystem, its integration into educational settings has gained significant momentum. Literature indicates that biomimicry-centered education positively impacts students‘ scientific process skills, creativity, and higher-order thinking (Harlow et al., 2023; Canbazoglu Bilici et al., 2021). Studies have reported that such applications support students' understanding of environmental concepts like sustainability and renewable energy (Staples, 2005) and foster a more conscious and sensitive perspective toward the environment (Sürgü and Güneş, 2022). Additionally, biomimicry contributes to self-reflection, innovative thinking (Alawad and Mahgoub, 2014; Soja, 2014), and spatial reasoning skills during design visualization (Zhu et al., 2024).

While research has spanned various educational levels -from university (Harlow et al., 2023) to preschool (Mart et al., 2024) - studies focusing on the high school level remain relatively limited and often confined to curriculum recommendations. This represents a significant research gap, as adolescence is a critical stage for the development of cognitive and abstract reasoning skills. A recent international review of biomimicry education studies further confirms this pattern: most published research has concentrated on undergraduate students, with only a small proportion of studies involving high school students (Yeter et al., 2023). It is also recommended that research be conducted in different age groups beyond middle schools in Türkiye (Uluçinar Sagir et al., 2022). Dawson (2007) highlighted this developmental disparity, noting that middle school students' knowledge levels regarding biotechnological applications are often lower than those of high school students, necessitating more focused research on the latter. To prepare future innovators of eco-friendly technologies, integrating biomimicry into projects and workshops is considered essential (Avci, 2019).

Since adolescence is a period in which abstract thinking develops, it is considered important to study the perception of biomimicry. According to Piaget's (1972) theory of cognitive development, adolescence marks the transition to the formal operational stage, in which individuals become increasingly able to think abstractly, reason hypothetically, and move systematically between different conceptual domains. More recent work on cognitive development has highlighted that adolescents' capacity for scientific reasoning and analogical transfer continues to develop throughout the high school years and is shaped by educational experience as well as by maturation (Zimmerman, 2007; Richland and Simms, 2015). For 9^th^-grade science high school students, who are at the beginning of the abstract thinking stage and follow a rigorous science-focused curriculum, biomimicry is considered to provide a particularly visible context in which these developing cognitive capacities can be observed.

At a more specific level, biomimicry can be considered a form of analogical reasoning, in which a biological system serves as a source domain and a design problem as a target domain. According to Gentner's (1983) structure-mapping theory, successful analogical transfer depends not on surface similarity, such as shape or appearance, but on the alignment of relational structure between the two domains that is, on identifying how the parts of a biological system function together and applying that relational pattern to a new context. From this perspective, biomimicry can be considered not simply a matter of observing nature but a cognitive task in which learners must extract the functional logic of a biological system and map it onto an engineering or design problem. Consistent with this view, recent empirical work has shown that one of the main difficulties students experience in biomimicry tasks is precisely this analogical step the transfer of knowledge from biology to technology or design (Yeter et al., 2023). Despite the conceptual fit between biomimicry and analogical reasoning, however, empirical work on how adolescents in academically high-achieving school settings actually engage in this nature-to-design transfer remains limited.

Despite its potential, biomimicry in education has primarily been explored through university students in science fields (Yildirim, 2019). There is a lack of focus on the perceptions of students in Science High Schools, a group of academically gifted students intensive in science education. Investigating the biomimicry perceptions of this specific group is vital for understanding how future scientific leaders bridge the gap between biological knowledge and technological design. While existing studies generally focus on the technical aspects of biomimicry, this study fills a gap in the literature by examining how adolescents perceive nature as a design partner and the potential impact of this perception on environmental ethics. In this context, the research was conducted at a science high school located within a grove, focusing on science education, and comprised of academically selected students, where students spend their breaks, lunch breaks, and free time. Accordingly, this study aims to examine science high school students' understanding of biomimicry, their ability to relate nature to daily life, and their capacity to translate these relationships into design processes. To this end, the study seeks to answer the following research questions:

1. What are the knowledge levels of science high school students regarding biomimicry and the characteristics of their time spent in nature?
2. How do these students relate natural structures and processes to daily life?
3. Can science high school students accurately match nature-inspired designs with their biological counterparts?
4. What are the characteristics of the biomimicry designs developed by these students?
5. What are the students' views on the overall importance of biomimicry?

## Methods

### Study design

In this research, we sought to identify the ability of science high school students to find solutions to daily life problems inspired by nature and to determine their nature awareness within the framework of the biomimicry approach. To achieve this, we conducted a qualitative case study. Throughout all stages of the research, we took into consideration the principles of the Standards for Reporting Qualitative Research (SRQR) (O'Brien et al., 2014).

### Participant

The study group consisted of 29 students (Female = 16, Male = 13) attending the 9^th^ grade at a science high school in Istanbul during the 2025–2026 academic year. We employed a purposive sampling method to select participants with the cognitive competence required to conceptualize the notion of biomimicry. In this context, students attending a science-intensive high school were preferred for the sample. We chose purposive sampling because it ensures that participants align more effectively with the research aims and objectives, thereby enhancing the rigor of the study and the reliability of the data (Campbell et al., 2020).

Prior to the study, we established the following inclusion criteria: (1) residing in Istanbul, Türkiye, (2) being enrolled in the 9th grade of a science high school, (3) having completed a preparatory year at the same institution, (4) the school being located within a forested area (grove), (5) possessing native-level proficiency in Turkish reading and writing, and (6) volunteering to participate in the research.

We selected 9^th^-grade students for two primary reasons. First, they possess the observation, expression, and abstract thinking skills necessary to comprehend the concept of biomimicry. Second, the concept of biomimicry is explicitly defined and supported with examples and visuals in the 9^th^ Grade Biology Textbook (https://ogmmateryal.eba.gov.tr) and the 9^th^ Grade Biology Workbook (2017–2023) (https://ogm-large-cdn.eba.gov.tr/ogm-materyal/calisma_defteri/f8/9/biyoloji/index.html) within the (Ministry of National Education, 2024) curriculum. Furthermore, the MoNE, (2024) 9^th^ Grade Biology Textbook (https://ogmmateryal.eba.gov.tr) covers topics such as Science, the Nature of Science, and Scientific Research Processes within the “Life” unit, encouraging students to engage in critical thinking regarding these concepts.

The students' contact with the grove is not a structured part of the curriculum and does not take place within scheduled lessons or planned outdoor activities. The grove forms the everyday physical environment of the school, where students spend their breaks, lunch periods, and free time.

### Recruiting participants and data collection

The study was approved by the Ethics Committee of Istanbul Gelişim University (Date: 30.05.2025, Decision No: 2025-11). Subsequently, official permission to conduct the research was granted by the Istanbul Provincial Directorate of National Education (Application No: MEB.TT.2025.030101.01) via an official letter dated 14.10.2025. All participants signed the Informed Consent Form.

We collected the data from the participating schools where the official permission procedures had been completed. The second author conducted face-to-face sessions with the participants, who were selected through purposive sampling, within their classroom environments. As all participants were proficient in reading and writing Turkish, the written semi-structured data collection forms were prepared and completed in Turkish. The second author explained the research objectives to the students and provided them with sufficient time to fill out the forms using pen and paper. The researcher remained with the participants until all forms were completed and answered any questions they had.

We conducted the data collection process individually with each participant. The number of participants was determined based on data saturation and representativeness (Guest et al., 2006). We began the coding process by examining each participant's form and concluded the data collection once we decided that the data could be categorized under specific themes and that saturation had been reached.

### Developing the data collection instrument

To identify the ability of science high school students to find nature-inspired solutions to daily problems and to determine their nature awareness, we developed a written semi-structured data collection form. Qualitative surveys with open-ended written responses are accepted in the literature as an independent qualitative data collection method, and this approach is considered suitable for studies that aim to examine how participants understand and explain a given concept (Braun and Clarke, 2013). In qualitative research, written accounts have been recognized as a legitimate and effective means of gathering rich descriptive data; written responses are often more focused and reflective than oral interview transcripts, and this can ease the analysis and interpretation process (Handy and Ross, 2005). This format was selected because biomimicry design is a reflective and creative process. It requires participants to observe nature, find functional similarities between living organisms and human-made objects, and develop their own design ideas. These cognitive activities require concentration and uninterrupted thinking, which is difficult to maintain under the time pressure of a verbal interview. It should also be noted that our procedure constitutes a researcher-administered qualitative survey, and (Braun and Clarke 2013, p. 135) describe this type of survey as essentially comparable to an interview. The second author was present during all sessions, explained the research aims, asked for further explanation when responses were short or unclear, and answered participants' questions. In this way, the procedure included the interactive elements of a face-to-face interview while preserving the reflective nature of written responses. This format was chosen for its capacity to encompass both closed and open-ended questions, positioning it formally between structured and unstructured instruments (Bryman, 2016). The questionnaire consists of four distinct sections:

- *Section 1:* Contains closed-ended questions to determine the demographic characteristics of the participants.
- *Section 2:* Focuses on the participants' ability to relate nature to daily life. Participants were presented with 12 nature-related terms and asked to identify which objects they encounter or use in daily life they relate these terms to, along with their reasoning. The objective here is to reveal the participants' capacity for knowledge transfer from nature to daily life.
- *Section 3:* Designed to evaluate matching skills and deepen the understanding of biomimicry. Students were asked to match six specific biomimicry designs with the organisms that inspired them. This section includes representative biomimicry designs that can be categorized according to top-down and bottom-up approaches (Biomimicry 3.8., 2015; Zari, 2007). Sections 2 and 3 collectively aim to build a foundational understanding of the biomimicry approach and prepare participants for the final design phase.
- *Section 4:* Comprises seven open-ended questions developed by adopting the bottom-up approach (Biomimicry 3.8, 2015; Zari, 2007). In the analysis of this section, student responses were coded based on the steps of the “Biology-to-Design Spiral” (Macnab, 2012). These questions were designed to elicit the participants' biomimicry design ideas and their qualitative reflections on the approach.

The development of the questions involved an extensive literature review covering biomimicry elements and design approaches (Purwaningsih et al., 2024; Biomimicry 3.8., 2015; Zari, 2007). Additionally, we analyzed the MoNE 9^th^ Grade Biology Textbook (Ministry of National Education, 2022, 2024) and the General Directorate of Secondary Education Workbook (2017–2023). To ensure the clarity and content validity of the instrument, we submitted the draft to a panel of five experts, including a child development specialist, a biologist, and a teacher. Based on expert feedback, explanatory statements were added in parentheses to Questions 2 and 5 in the fourth section of the data collection tool. Question 2 was revised as follows: “Which characteristics of the organism you chose caught your attention? (e.g., movement, habitat, feeding habits, defense mechanisms, structure, etc.)” and Question 5 was revised as follows: “In which field does your designed product serve? (e.g., transportation, health, technology, energy, architecture, etc.).” To verify the comprehensibility of the questions using a data collection tool modified with expert feedback, we conducted a pilot study with five randomly selected high school students (outside the main sample). The final questionnaire is presented in Appendix A.

### Researchers backgrounds

The research team consists of academic personnel specialized in the field of child development, with extensive experience working with children aged 0–18. The third author serves as the coordinator of a university-affiliated children's science center, and the first author operates a science school under this coordination. Together, they conduct multidisciplinary studies in collaboration with various scientific branches.

We remained consciously aware of the potential influence of our professional backgrounds on the research process. As recommended by Creswell and Creswell (2017), we took deliberate steps to minimize potential biases during the data collection and interpretation phases. To ensure the accuracy of the findings and to fully comprehend the students' expressions, the authors maintained constant communication and collaborative dialogue while interpreting the forms completed by the participants.

### Rigor

To ensure the integrity and reliability of our research, we adhered to the standards set by (O'Brien et al. (2014) and the criteria established by Lincoln and Guba (1985), 2016). With 29 participants in the study, the second author allowed all participants sufficient time to complete the forms, which supported *credibility (internal validity)*. When participants found questions unclear, the researcher provided explanations and concrete examples to ensure a common understanding. The depth of the data was further supported by the participants‘ own responses: most students answered the open-ended questions in Section 4 with several sentences and provided enough detail for thematic analysis. Data saturation was checked through a concurrent coding process: the research team began analyzing the forms of the initial participants while data collection was still ongoing, and continued until no new themes or categories emerged across subsequent responses. A small number of questions in the earlier sections were left blank by some participants, but all participants completed every open-ended question in Section 4, which constituted the main analytical focus of the study.

To strengthen *transferability (external validity)*, we established detailed inclusion criteria during the research planning phase and strictly adhered to these parameters. During the coding process, we assigned a pseudonym to each participant to ensure anonymity and presented the findings accordingly.

To verify *dependability (reliability)*, we developed the data collection instrument based on extensive literature, biomimicry elements, and design approaches (Purwaningsih et al., 2024; Biomimicry 3.8., 2015; Zari, 2007), as well as the MoNE 9^th^ Grade Biology Textbook (2022; 2024) and the General Directorate of Secondary Education Workbook (2017–2023). We proceeded by validating the codes concurrently. The second author transcribed all data into a digital format while preserving the participants' original expressions, and the first author cross-checked these transcriptions. The qualitative analysis process was finalized through cumulative and regular meetings held by all three authors. All coding decisions were recorded and tracked within MAXQDA24, which provided an audit trail of every code, category, and theme. This audit trail allows the analytical process to be retraced step by step and supports the transparency of the analysis.

To support *confirmability (objectivity)*, we paid particular attention to the role of the research team in the interpretation process. During the analysis, each author was free to question another author's interpretation of any segment, and a code or theme was retained only when all three authors agreed on it. This shared decision rule helped ensure that no single researcher's perspective could dominate the final coding.

Member checking, in the form of returning interpreted data to participants for confirmation, was not considered necessary in the present study because participants produced their own written responses directly. Since the analysis is based on the participants‘ own words rather than on transcriptions or paraphrased summaries by the researchers, the risk of misrepresenting participants' meanings during data capture is reduced. The trustworthiness of the analysis was instead supported through the triangulation procedures described above.

## Data analysis

To ensure a deep and comprehensive understanding of the data, all three authors independently reviewed the transcribed materials before initiating the formal analysis. We performed content analysis using the MAXQDA24 software, where the thematic coding process involved identifying similarities and differences among the emerging codes to organize them into cohesive themes. This coding procedure followed a multi-stage collaborative approach. The second author first coded all forms and developed an initial set of codes and categories. The first and second authors then held a series of meetings in which they went through the coded data segment by segment, discussed the meaning and naming of each code, and agreed on the final code labels and the content covered by each code. The level of agreement between the two authors was high, and the few cases where their interpretations differed were resolved through discussion until both authors reached the same understanding. A final round of review was conducted with the third author to confirm the themes, categories, and naming conventions (nomenclature), which are presented in the findings through both frequencies and qualitative content descriptions. The findings are organized in a sequence that follows both the structure of the data collection instrument and the analytical framework of the study.

The questions in the fourth section of the data collection instrument were developed based on the bottom-up biomimicry design approach (Biomimicry 3.8., 2015; Zari, 2007). To systematically analyze the student responses in this section, we utilized the stages of the “Biology-to-Design Spiral” as our primary analytical framework. Accordingly, we coded the responses into the stages of discovery, abstraction, brainstorming, emulation, and evaluation (Macnab, 2012). Within this framework, the discovery phase included responses to the first and second questions regarding the selected organisms and the reasons for their selection, while the abstraction phase focused on the fourth question examining the specific problems the designs aimed to solve. The brainstorming phase encompassed the third question's focus on core design ideas, whereas the transformation of these ideas into concrete applications was evaluated within the emulation phase. Finally, the evaluation phase categorized responses to the fifth and sixth questions, which addressed the service areas of the designs and their environmental sustainability characteristics.

### Findings

#### Awareness of the biomimicry concept and affinity with nature

To identify the ability of high school students to find nature-inspired solutions to daily life problems and to determine their nature awareness within the framework of the biomimicry approach, we first gathered data regarding the participants' knowledge levels of the biomimicry concept and their habits of spending time in nature. These findings, which establish the foundational context for the study's subsequent design phases, are presented in Table 1.

**Table 1:** Participants' knowledge of the concept of biomimicry and characteristics of time spent in nature.

Categories	Codes	*n*	Categories	Codes	*n*
Awareness of the biomimicry concept	Not heard	6	Time spent in nature in the last month	Almost every day	18
	Heard	23		2–3 days a week	7
Time spent in nature in a day	Less than 15 min	5		1 day a week	4
	15–30 min	7	Definition of biomimicry	Nature-inspired design through imitating nature	10
	30–60 min	4		Design inspired by the characteristics of living things	11
	1–2 h	4		Related to biology	4
	More than 2 h	9		No knowledge	4

As illustrated in Table 1, the majority of the participants had previously encountered the concept of biomimicry and provided accurate definitions. For instance, the student coded F1 demonstrated conceptual clarity by defining biomimicry as “*human designs inspired by the structures or movements of biological organisms*.” Emphasizing the functional aspect of the approach, student F11 described it as “*making life easier by adapting the innate characteristics of living things into technology*.” Furthermore, student F22 reflected their perspective on nature while defining the concept, stating that biomimicry is “*creating human-made products by imitating the perfect order and functioning in nature*.”

Furthermore, we observed that the majority of participants have the opportunity to spend time in nature almost every day, thanks to the grove located within their school campus, with most spending more than 30 min daily in this environment. Most participants (23 of 29) had encountered the concept of biomimicry prior to the study, and only a small number (4 of 29) reported having no knowledge of the term. The definitions provided clustered into two complementary framings one centered on “imitating nature” and the other on “the characteristics of living things” while a smaller group framed biomimicry simply as a topic “related to biology.” All participants reported spending time in a natural setting at least once a week, and approximately half (13 of 29) spent more than an hour in nature on a typical day. This consistent interaction with a natural setting is considered to provide a significant observational basis for the students' engagement with biomimicry-based design processes.

#### Relational thinking: matching natural elements with daily life objects

The findings regarding the participants' ability to relate 12 different nature-based terms to designs encountered in daily life are presented in Table 2.

**Table 2:** Relational mapping from nature to daily life.

No	Nature term	Which daily life design does it resemble?	Why did you think so?
1	Sun	Light source products Heat source products Sunscreen	Light-emitting property Heating property Protection property
2	Wind	Air-emitting products Wind-powered products Electricity	Air-emitting property Energy production property Unanswered
3	Rain	Fixture/faucet Irrigation tool Water dispenser Cloud	Wetting property Dripping/flowing property Sound/noise similarity Functional similarity Unanswered
4	Cactus	Sharp/piercing tools Water storage products Ornamental potted plant Desert Clothing Unanswered	Sharp/piercing property Water storage property Self-protection property Establishing a connection Unanswered
5	Tree Roots	Carriers Foundation Tools and equipment Schema/concept map Food source Life Unanswered	Shape/appearance similarity Functional similarity Being a source of life Unanswered
6	Stone/Rock	Construction materials Weight Tools and equipment Art Bridge Unanswered	Shape, appearance and space similarity Being heavy property Hard/durable property Functional similarity Art Unanswered
7	Bird	Flying products Sharp/piercing tools Pets	Functional/appearance similarity Having wings Being a pet
8	Hedgehog	Sharp-structured tools Cactus Comb varieties Evening Game materials Defense tools Sponge Unanswered	Functional similarity Sharp/thorny structural property Morphological similarity Establishing a connection Unanswered
9	Chameleon	Camouflage Color-changing products Television Tools and equipment Unanswered	Color-changing property Camouflage property Physical similarity Establishing a connection
10	Spider Web	Thread, tie, weave Chain, rope Tools and equipment Connection network Fractals Character	Durability property Weaving property Appearance, structure, functional similarity Unanswered
11	Crab	Pincer-like products Tools and equipment Robotic arm Metro Zodiac sign	Similarity to pincers Strength property Side-walking property Establishing a connection Unanswered
12	Elephant	Vehicle Irrigation tool Tools and equipment Elephant skin Unanswered	Carrying property Functional similarity Area of use Physical similarity Unanswered

When we analyzed the students' ability to relate 12 different natural terms to daily life objects, we found that the highest level of consensus was in the “*Bird-Flying products*” codes. This was followed by highly consistent responses in the categories of “*Wind-Air-emitting products*” and “*Sun-Light source products*.” In contrast, the natural element that students matched with the widest variety of products was “*Tree roots*.” The responses in this category were distributed among “*Carriers, Foundation, Tools and equipment, Schemas/Concept maps, Food source*, and *Life*.” Additionally, while a very limited number of students were unable to find a related object or explain the reasoning behind their match, the vast majority of participants successfully identified objects from their daily lives for each natural term.

Examining the students' justifications for these pairings reveals significant functional insights. For instance, regarding “*Tree roots*,” student F18 used a “*Cable*” analogy, stating, “*I compared them functionally because cables provide energy and tree roots provide nutrients to the tree*.” For “*Spider web*,” student F17 suggested a “*Tape*” analogy, noting, “*When something sticks to a spider web, it remains suspended there; I compared them based on this property*.” Furthermore, student F10 drew a parallel between “*Rain*” and a “*Showerhead*,” explaining that “*the way showerheads emit water resembles rain*.”

Most students grounded their analogies in functional relations (e.g., “carrying”, “emitting”, “sticking”) rather than in surface attributes such as color or shape. The term “Tree roots” produced the widest range of daily-life analogs (six distinct categories), while “Bird” produced the most uniform mapping, with students converging on a single functional category.

#### Functional analysis of biomimicry designs

In this stage of the study, we presented the participants with specific pairings of organisms found in nature and their corresponding objects in daily life, subsequently asking them to describe which biological characteristics they believed inspired these designs. The detailed descriptions and functional contexts of the biomimicry examples provided to the students are presented in Table 3.

**Table 3:** Matching nature-inspired designs with biological sources.

No	Organism in nature	Matched design	Design approach adopted	From which characteristic of the organism do you think it was inspired?
1	Dragonfly	Helicopter	Top-down	Inspiration from its flight property Inspiration from its wings Inspiration from its aerodynamics
2	Kingfisher	Train	Top-down	Inspiration from its beak Inspiration from its speed Inspiration from its aerodynamics
3	Termit/Ant Mound	Eastgate Building thermal control system	Top-down	Inspiration from its nesting property Inspiration from its architectural structure Inspiration from its system property
4	Lotus Flower	Paint	Bottom-up	Inspiration from its dirt-repellent property Inspiration from its color/pigmentation Inspiration from its ability to stay afloat Inspiration from its structure
5	Burdock Plant	Velcro	Bottom-up	Inspiration from its adhesive property Inspiration from its thorny structure
6	Sunflower	Solar panels	Top-down	Inspiration from its heliotropic (sun-tracking) property Inspiration from its solar energy absorption

As presented in Table 3, the participants identified various sources of inspiration for the pairing of organisms and daily objects, most notably mentioning “*wings*” for the *Dragonfly-Helicopter*, “*beak*” for the *Kingfisher-Train*, and “*architectural structure*” for the *Termite mound-Eastgate Building* thermal control system. Additionally, students highlighted “*color*” for the *Lotus flower-Paint*, “*adhesion*” for the *Burdock plant-Velcro*, and the “*heliotropic property*” and “*turning toward the sun*” for the *Sunflower-Solar panels*. While a few instances of non-response were observed, the vast majority of students successfully established these relationships and made accurate functional inferences.

We selected the biomimicry design examples included in the matching section of the instrument to represent both top-down and bottom-up design approaches. In this context, we categorized the *helicopter* (inspired by the dragonfly), the *high-speed train* (inspired by the kingfisher's beak), the *Eastgate Building's thermal control system* (utilizing the natural ventilation of termite mounds), and *solar panels* (developed from the sunflower's sun-tracking behavior) as examples of the top-down design approach. Conversely, we included *self-cleaning paints* (developed by examining the water-repellent properties of the lotus leaf surface) and *Velcro* (inspired by the attachment mechanism of burdock seeds) as representative examples of the bottom-up design approach.

When explaining the logic behind these designs, student F2 articulated the biological form's solution to an engineering problem (noise and speed) regarding the *Kingfisher-Train* relationship, stating, “*Due to the structure of its beak, this type of bird does not make noise when diving into the water; the front-end design of trains might have been influenced by this*.” Regarding the *Termite mound-Building* relationship, student F17 expressed how it offers a solution to engineering design in construction systems, noting, “*The presence of many hotel rooms in every corridor might have been inspired by the many ant homes in every corridor*.”

Across the six design organism pairs, participants identified inspiration sources of two qualitatively different kinds: for animal-based pairs (e.g., Dragonfly-Helicopter, Kingfisher-Train), students most frequently named visible anatomical features such as *wings* or *beaks*, while for plant-based pairs (e.g., Lotus-Paint, Burdock-Velcro), they more often named functional properties such as “*dirt-repellent property*” or “*adhesive property*”. Non-responses were observed for only a small number of items.

#### Student designs according to the biology-to-design spiral

We asked the students to develop an original design by responding to a series of guided prompts: (1) Choose an organism from nature; which one did you select? (2) Which characteristics of this organism caught your attention (e.g., movement, habitat, feeding habits, defense mechanisms, structure)? (3) Inspired by these features, what kind of product would you design to solve a specific problem? (4) Describe in detail how your design addresses this problem. (5) In which field would your product serve (e.g., transportation, health, technology, energy, architecture)? (6) What considerations would you prioritize to ensure this design is eco-friendly and sustainable? (7) Why do you believe it is important to take inspiration from nature to solve daily life problems?

To systematically analyze the participants‘ responses, we categorized their answers according to the dimensions of the Biology-to-Design Spiral, specifically the stages of discovery, abstraction, brainstorming, emulation, and evaluation. This structured analysis, which maps the students' cognitive journey from biological observation to sustainable design solutions, is presented in Table 4.

**Table 4:** Development of nature-inspired eco-friendly/sustainable designs for daily life problems.

Discovery (chosen organism)	Discovery (characteristic)	Abstraction (problem solved)	Brainstorming & emulation (design)	Evaluation (field of service)	Evaluation (eco-friendly)
Reptiles	Natural ability	Health problems	Technological tool	Health	Use of recycled materials
Birds	Social domains	Environmental problems	Tools and equipment	Transportation	Use of sustainable materials
Fish	Being strong	Defense industry problems	Performance-enhancing tools	Defense industry	Renewable energy
Mammals	Mobility	Exploration problems	Security and defense tools	Technology	Species conservation
Arthropods	Calmness	Security problems	Robot and AI tools	Architecture and engineering	Unanswered
Mollusks	Intelligence	Excessive cost problems			
Human	Cuteness	Increasing effectiveness of tools			
	Self-resemblance	Energy and insulation problems			
	Self-protection/defense	Social life problems			
		Unanswered			

We evaluated the participants' responses in accordance with the stages of the Biology-to-Design Spiral.

### Discovery

When we examined the students' responses regarding their chosen organisms and their specific traits within the discovery phase of Table 4, we observed that the vast majority preferred mammals. This was followed by fish, reptiles, birds, arthropods, mollusks, and humans, respectively. These findings suggest that within the context of biomimicry, students tend to focus on organisms they have the opportunity to observe in daily life and whose functional characteristics they are familiar with. Furthermore, all participants except for one selected their subjects from the animal kingdom. Regarding the characteristics of these organisms, students most frequently emphasized the theme of “natural abilities.” Other highlighted traits included social domains, strength, mobility, self-protection/defense, intelligence, cuteness, calmness, and self-resemblance. These results indicate that students primarily conceptualize biomimicry through structure- and function-based characteristics.

### Abstraction

In the abstraction phase, we analyzed the participants' responses to the question investigating which problems their designs aimed to solve. Solutions for health-related issues ranked first, followed by challenges in the defense industry, security concerns, efforts to increase the effectiveness of tools and equipment, social life issues, environmental problems, exploration-related challenges, and energy/insulation problems. A small number of participants did not provide a response to this question.

### Brainstorming and emulation

When we evaluated the responses aimed at identifying a design idea (brainstorming) and transforming that idea into a concrete application (emulation), technological devices and security/defense tools became prominent. Other design types included performance-enhancing tools, general equipment, and robotics or artificial intelligence-based tools. This finding demonstrates that students strongly associate biomimicry with technology- and engineering-based solutions.

### Evaluation

In the evaluation phase, we examined the service areas of the students' designs and their eco-friendly characteristics. The results show that the developed designs were most concentrated in the fields of architecture and engineering, followed by transportation, health, the defense industry, and technology. This distribution reveals that students view biomimicry as an interdisciplinary tool for problem-solving. Finally, regarding the environmental aspects of the designs, we found that students primarily prioritized the use of recycled materials, followed by sustainable material use, renewable energy, and species conservation. Some students did not respond to this question. These findings suggest that while students evaluate biomimicry within the framework of environmental sustainability, their focus on the nature conservation dimension remains relatively limited.

**Figure 1:**
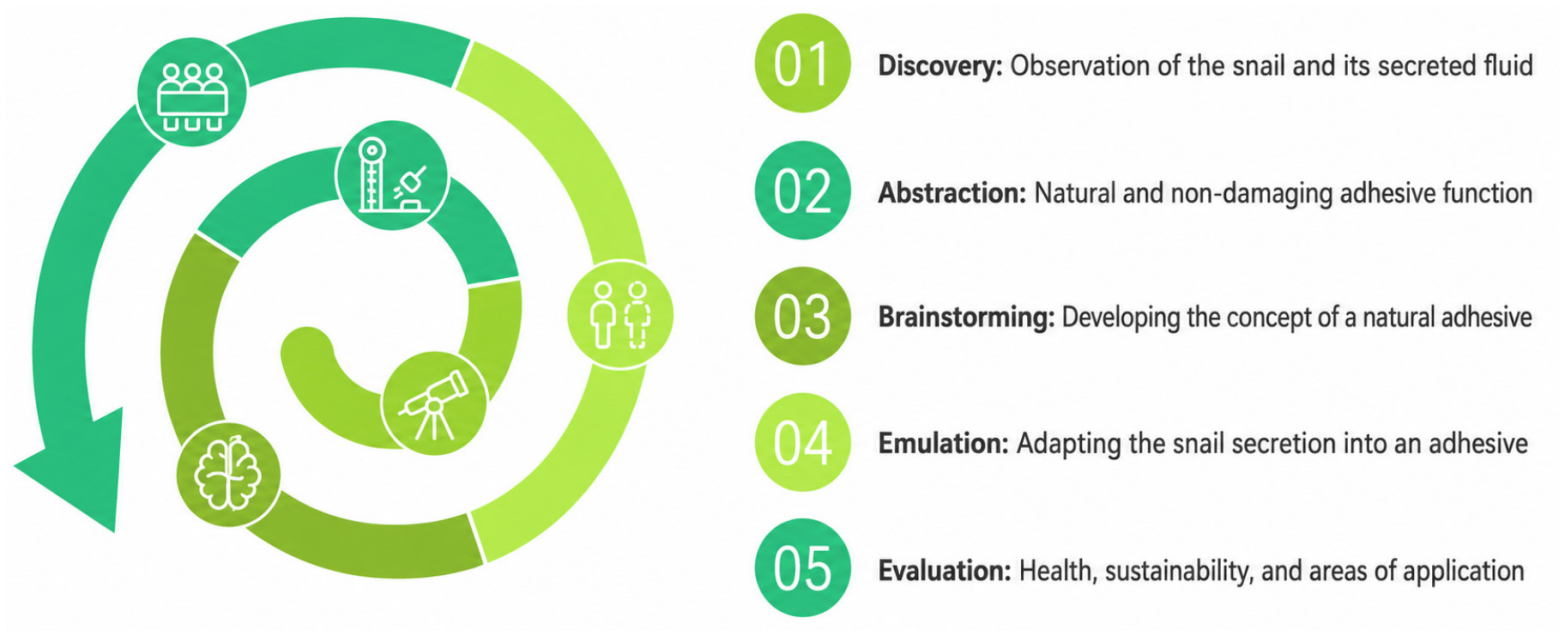
Adaptation of student F3′s design process to the biology-to-design spiral by the researchers (Macnab, 2012).

When Figure 1 is examined, it is observed that student F3 selected the snail as the biological model. The student stated that the snail was chosen due to its shape and the fluid it secretes, and inspired by these traits, they could design an adhesive. It was specified that the designed adhesive would be natural and healthy, and the materials used in its content would be eco-friendly and sustainable. In this context, the observation of the snail and its secretion was classified as discovery; the natural and non-harmful bonding function as abstraction; the development of the natural adhesive idea as brainstorming; the adaptation of the snail secretion to an adhesive as emulation; and the aspects of health, sustainability, eco-friendliness, and fields of application as evaluation.

### Students' views on the importance of biomimicry

When the responses to the seventh question of this section -which investigated why it is important to take inspiration from nature to solve daily life problems- were analyzed, it was seen that the majority of students stated that “*nature offers ready-made solutions*.” Others touched upon themes such as “nature-inspired solutions providing convenience and efficiency,” “nature being a source for generating new ideas,” and “the continuity of life and species.” Examining specific student statements, F15 emphasized that “*instead of spending energy, money, and effort on R&D studies to solve problems, easier solutions should be produced by taking inspiration from nature*.” Student F1 expressed a belief in the convenience provided by this approach, stating, “In nature, everything progresses within an order, and the probability of success increases when people take inspiration from already existing solutions for their problems.” Furthermore, F6 noted that a solution exists for every problem in nature, remarking, “*Because if we take inspiration from nature, we can find a solution to the problem we are experiencing more easily. Since we are a part of nature, we can find an answer to all questions*.”

## Discussion

This study, conducted to determine the nature-inspired problem-solving skills and nature awareness of high school students within the framework of the biomimicry approach, was carried out with students from a science high school located within a grove in Istanbul. These students possess high-level academic skills and undergo an intensive curriculum focused on preparatory year language training and science-mathematics education. The findings indicate that the participants had prior knowledge of the concept of biomimicry and provided definitions closely aligned with the scientific framework. Due to their educational background, most students focused on approaches such as imitating nature, developing nature-inspired designs, and creating designs based on the characteristics of living organisms. While the results reveal that students perceive biomimicry as a tool that facilitates life, the primary emphasis remains on integrating the functional traits of organisms into human life. Science high schools in Istanbul, which aim to identify and train gifted students as future scientists, provide an environment where students encounter scientific thinking and nature-inspired concepts. The ability of these students to define biomimicry can be linked to the “biomimicry” content integrated into the secondary biology curriculum and materials. The inclusion of biomimicry within the “World of Living Things and Their Characteristics” unit in the Ministry of National Education (2018) 9^th^-grade biology workbook, along with activities encouraging nature-inspired thinking, likely contributed to this familiarity. It is believed that the nature-oriented approaches of individuals in the students‘ immediate environment, the physical location of the school in a grove, and the teachers' tendency to encourage students to interact with nature and provide suitable learning environments all shape a child's approach to nature. Indeed, greener school surroundings and regular nature experiences have been associated with stronger learning outcomes and engagement in students (Kuo et al., 2019). Furthermore, cognitive development levels during adolescence, particularly the advancement of abstract thinking and analogical reasoning (Zimmerman, 2007; Richland and Simms, 2015), facilitate the understanding of such complex nature–design relationships. Literature also emphasizes that students can relate biological processes to problem-solving and develop conceptual awareness in educational contexts (Staples, 2005; Alawad and Mahgoub, 2014).

The second and third sections of the instrument aimed to establish a foundation for the biomimicry approach and prepare participants for the design phase in the fourth section. In the relational mapping section, we observed that students focused more on function than on form or shape when matching natural terms with daily objects. Their ability to relate tree roots to building foundations or rain to showerheads through a functional lens demonstrates an engineering-oriented perspective on biological systems. Similarly, Sumrall et al. (2018) found that students drew camouflage designs inspired by the color-changing properties of chameleons. These findings demonstrate that students are aware of the functions of living things in nature and understand how nature's natural processes provide solutions to problems. It was also found that students, in developing designs inspired by nature, were influenced not only by physical similarities but also by functional similarities. Most students successfully matched nature-inspired designs with their biological sources, a process that forms the basis of understanding biomimicry. Sanne et al. (2019) used similar examples -such as sharkskin-inspired swimsuits, Velcro, and self-cleaning surfaces- to enhance participants' interpretation of the biomimicry approach, a finding supported by Sumrall et al. (2018). Developing biomimicry-based designs requires a close understanding of nature and the roles and functions of living organisms within their natural habitat. The fact that the students participating in the study correctly identified which organism and which characteristic of that organism influenced the biomimicry design examples included in the data collection tool demonstrates their awareness of the functional characteristics of living things in nature. This reveals that the students not only observed nature but also understood the adaptation processes and problem-solving mechanisms of living organisms. This pattern, where students preferred functional relations over surface appearance when connecting nature to design, is also consistent with structure-mapping theory, which identifies the matching of relational structures between two domains as the core of mature analogical reasoning (Gentner, 1983). Regarding the selection of organisms, responses were largely limited to the animal kingdom, with only one student mentioning humans. This parallels the findings of Güller et al. (2019), which showed that children primarily conceptualize the “living” concept through animals. The finding that students' selections were almost exclusively limited to the animal kingdom, with a notable absence of plant-based models, underscores a common phenomenon in biology education known as “plant blindness” (Achurra, 2022). Students expressed being influenced by the functional traits of their chosen organisms, aligning with Zhu et al. (2024), who determined that children effectively use spatial thinking during analogy-based design processes. The preference for designing technological devices and defense-related tools in our study echoes the work of (Yakişan and Velioglu 2019), who noted a prominence of defense, power, and control themes in student-designed technological products inspired by animals. The notable inclination of participants toward defense, power, and security-themed designs can be attributed to the specialized academic context of Science High Schools. These institutions prioritize an intensive STEM curriculum that emphasizes engineering problem-solving and strategic innovation. Consequently, these high-achieving students may perceive biomimicry not only as a tool for environmental harmony but also as a mechanism for developing high-performance solutions to global security and technological challenges. This “engineering-centric” perspective likely stems from their intensive exposure to competitive scientific environments, which fosters a mindset focused on robustness and efficiency in design ideation. Furthermore, student F3′s design process, which transformed the observation of snail secretion into a sustainable adhesive, exemplifies the successful application of the Biology-to-Design Spiral (Discovery, Abstraction, Brainstorming, Emulation, and Evaluation). The focus on recycling, renewable energy, and zero-waste reflects a significant level of sustainability consciousness for this age group (Barrera-Hernández et al., 2020).

The findings demonstrate that students predominantly aim to generate solutions for real-world problems in health, transportation, architecture, and technology. This supports the potential of biomimicry to transform abstract biological knowledge into concrete, functional designs, an approach that Fried et al. (2020) argue increases students' tendencies to use biological information for social benefit. Consequently, biomimicry serves as a tool that enables students to not only learn biological concepts but also to apply them in meaningful, real-world contexts. This finding can be interpreted as related to the students being in adolescence. Adolescence is a developmental period in which individuals become more sensitive to environmental issues, current events, and social problems, and their tendency to think about and generate ideas on social issues increases. Therefore, the students' focus on social and environmental problems may stem from the developmental characteristics of adolescence. Furthermore, during this period, individuals may be more willing to express their opinions on social issues, develop solutions, and feel a sense of social responsibility. At the same time, the narrow range of organisms students chose and their concentration on technology-related applications suggest that everyday contact with nature does not, on its own, broaden adolescents' design imagination; structured classroom activities that follow the stages of the Biology-to-Design Spiral may therefore be needed to widen the kinds of organisms and problems they engage with. Evidence from environmental education research also indicates that such structured interventions can effectively support young people's connection to nature (Barrable and Booth, 2020).

## Conclusion

This study, involving 9^th^-grade science high school students in Istanbul, Türkiye, concludes that participants have a strong awareness of biomimicry and a frequent interaction with nature due to their school's location. While this exposure is not part of the formal curriculum, the daily presence of a natural environment within the school grounds appears to provide a meaningful observational background for these students, particularly in the context of an otherwise highly urbanized setting. A positive outcome of the research is that students perceive nature as a “mentor” rather than just a resource. Their ability to transition from biological knowledge to design logic is evident in their focus on function over form. Moreover, their emphasis on eco-friendly designs indicates an understanding of the philosophy of biology and its successful transfer into engineering design.

Beyond these specific findings, the study contributes to the biomimicry education literature in two ways. First, the present study focuses on academically high-achieving science high school students, and it provides empirical evidence that 9^th^-grade students in this group can move through the stages of the Biology-to-Design Spiral and produce coherent nature-inspired design ideas. Second, the study offers a detailed picture of how adolescents combine functional thinking, environmental sensitivity, and an interdisciplinary orientation (covering health, transportation, architecture, defense, and technology) when generating their own biomimicry designs.

In practical terms, the findings support a more structured place for biomimicry within science high school education. Biology lessons could include short, design-oriented activities in which students follow the stages of the Biology-to-Design Spiral, starting from the observation of an organism and ending with a sustainable design idea. Such activities could help students see nature not only as a topic of biological knowledge, but also as a source of solutions for real-life problems.

## Limitations

Our research has several limitations, each of which points to a direction for future research. Primarily, the study group is limited to 1^st^-year volunteers at a science high school located in a grove in Istanbul, Türkiye. Future studies could compare biomimicry perceptions across different types of high schools and across schools located in different physical settings, in order to examine how the school context shapes adolescents‘ design thinking. Secondly, the data are restricted to the instruments developed through literature review and expert opinion. Mixed-methods designs that combine the present qualitative protocol with quantitative measures could provide a more complete picture of how biomimicry perceptions develop. Finally, our findings are limited by the paradigms of qualitative research, which does not aim for broad generalizability. Cross-cultural comparative studies, in which similar protocols are applied to gifted adolescents in different countries, would help clarify which patterns are tied to the Turkish educational context and which reflect more general features of adolescents' engagement with biomimicry.

## Data Availability

The raw data supporting the conclusions of this article will be made available by the authors, without undue reservation.
