# Supplementary material for: Eco-friendly design inspired by nature: examining adolescents' perceptions of biomimicry
Source: Front Psychol. 2026 Jun 30;17:1833615. doi: 10.3389/fpsyg.2026.1833615 (PMC13363333; doi:10.3389/fpsyg.2026.1833615)
Supplement: Supplementary file 1 [file Supplementary_file_1.DOCX]

**APPENDIX A: DATA COLLECTION INSTRUMENT**

**SECTION 1: RELATING NATURE TO DAILY LIFE**

In this section, you are asked to consider the functions of the natural elements listed in the table below and write which designs you see or use in daily life they resemble, along with your reasoning. Please do not leave any fields blank.

| **No** | **Nature Term** | **Which daily life design does it resemble?** | **Why do you think so?** |
| --- | --- | --- | --- |
| **1** | **Sun** |  |  |
| **2** | **Wind** |  |  |
| **3** | **Rain** |  |  |
| **4** | **Cactus** |  |  |
| **5** | **Tree Roots** |  |  |
| **6** | **Stone/Rock** |  |  |
| **7** | **Bird** |  |  |
| **8** | **Hedgehog** |  |  |
| **9** | **Chameleon** |  |  |
| **10** | **Spider Web** |  |  |
| **11** | **Crab** |  |  |
| **12** | **Elephant** |  |  |

**SECTION 2: MATCHING NATURE-INSPIRED DESIGNS**

In the tables below, organisms found in nature and products designed by taking inspiration from certain characteristics of these organisms are presented in a mixed order. In this section, you are asked to match the organisms in Table 1 with the designs in Table 2. After the matching process, you are also expected to write which specific characteristic of the organism served as the inspiration for that design.

**Table 1**

| **No** | **Organism in Nature** | **Matched Design** | **From which characteristic of the organism do you think it was inspired?** |
| --- | --- | --- | --- |
| **1** | **Dragonfly 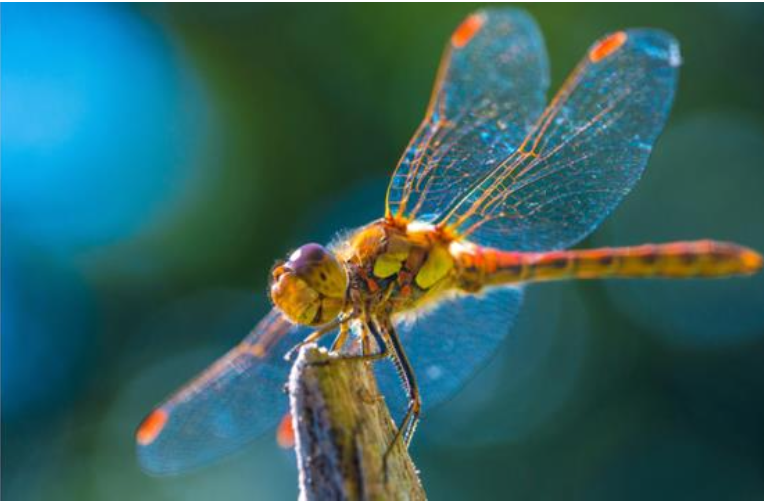** |  |  |
| **2** | **Kingfisher 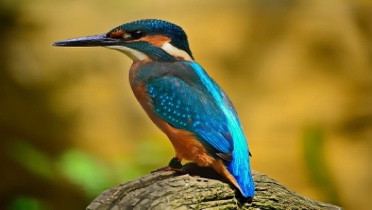** |  |  |
| **3** | **Termite/Ant Mound 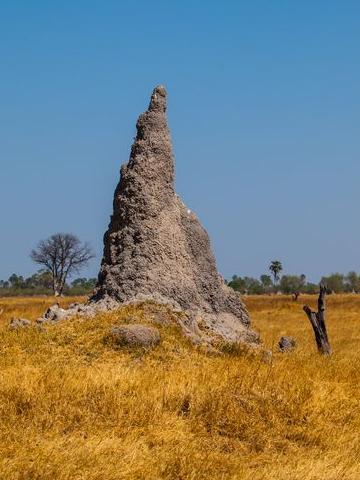** |  |  |
| **4** | **Lotus Flower 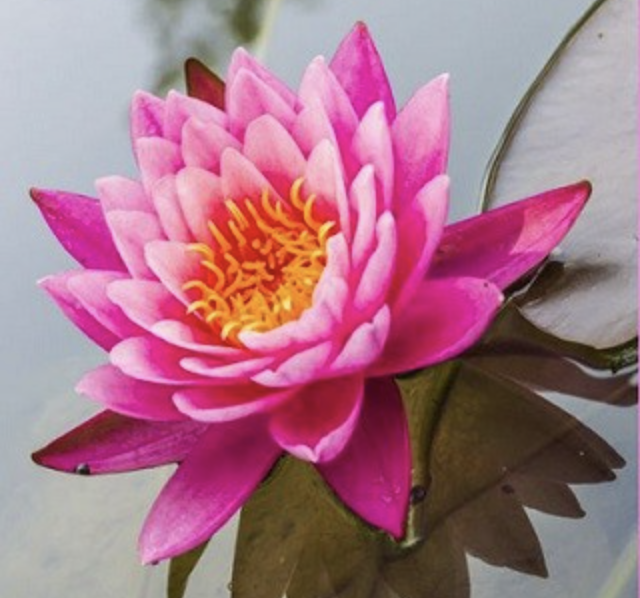** |  |  |
| **5** | **Burdock Plant 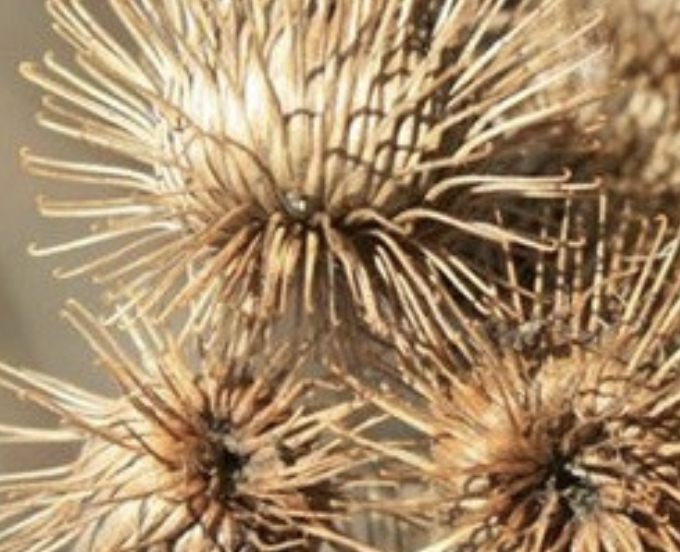** |  |  |
| **6** | **Sunflower**  **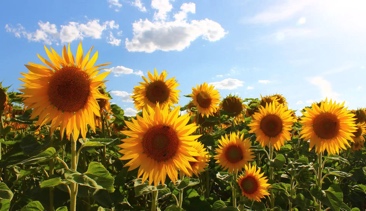** |  |  |

**Table 2**

| **No** | **Design** |
| --- | --- |
| **A** | **Velcro**  **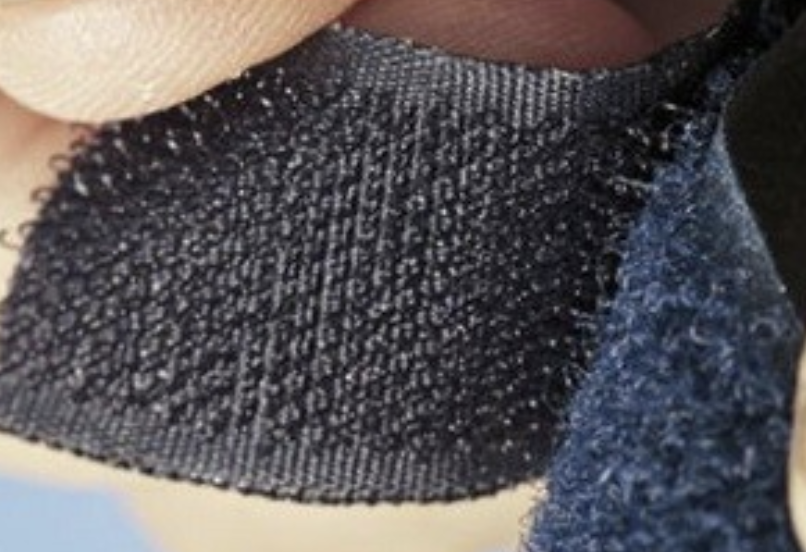** |
| **B** | **Building**  **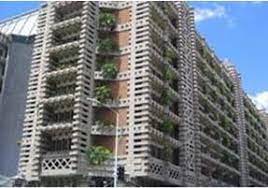** |
| **C** | **Paint (Self-cleaning/Dust-repellent) 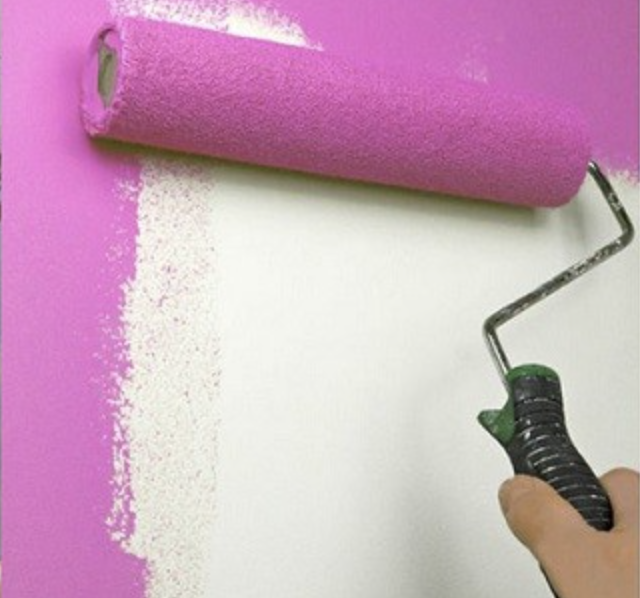** |
| **D** | **Train**  **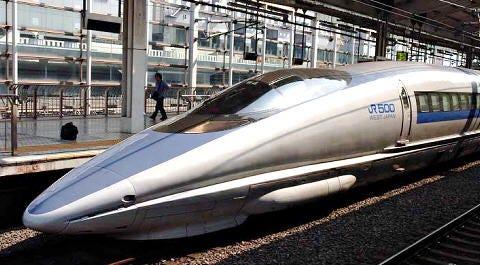** |
| **E** | **Solar Panels**  **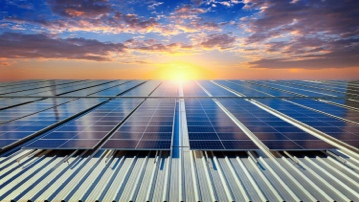** |
| **F** | **Helicopter**  **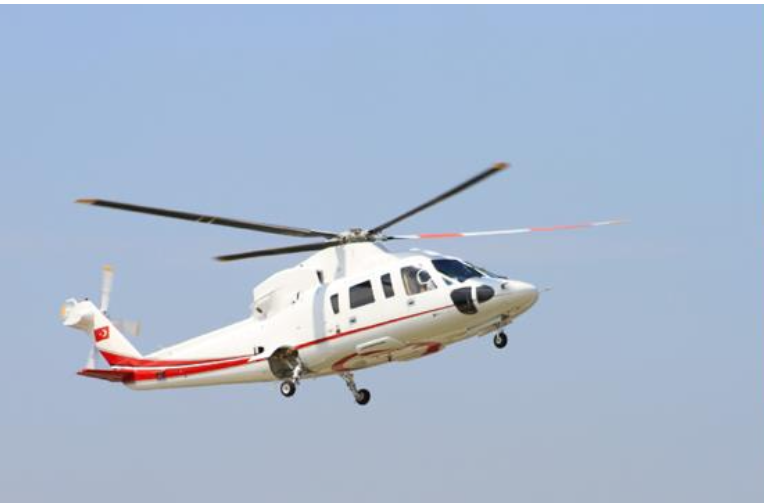** |

**SECTION 3: DEVELOPING ECO-FRIENDLY / SUSTAINABLE DESIGNS INSPIRED BY NATURE FOR DAILY LIFE PROBLEMS**

In this section, you are asked to provide detailed answers to the following questions based on your own ideas.

1. **Think of an organism from nature. Which organism did you choose?**
2. **Which characteristics of the organism you chose caught your attention? (Movement, habitat, feeding habits, defense mechanism, structure, etc.)**
3. **Inspired by these features, what kind of product would you design to solve a problem?**
4. **Does your design bring a solution to a specific problem? Please explain in detail.**
5. **In which field does your designed product serve? (e.g., transportation, health, technology, energy, architecture, etc.)**
6. **What would you pay attention to in order to make this design eco-friendly / sustainable?**
7. **In your opinion, why might it be important to take inspiration from nature to solve daily life problems?**
